# Construction of Highly Ordered Glyco‐Inside Nano‐Assemblies through RAFT Dispersion Polymerization of Galactose‐Decorated Monomer

**DOI:** 10.1002/anie.202015692

**Published:** 2021-03-25

**Authors:** Liang Qiu, Haoran Zhang, Thomas Bick, Johannes Martin, Petra Wendler, Alexander Böker, Ulrich Glebe, Chengfen Xing

**Affiliations:** ^1^ Key Laboratory of Hebei Province for Molecular Biophysics Institute of Biophysics Hebei University of Technology Tianjin 300401 P. R. China; ^2^ Department of Life Science and Bioprocesses Fraunhofer Institute for Applied Polymer Research IAP Geiselbergstr. 69 14476 Potsdam-Golm Germany; ^3^ Department of Biochemistry University of Potsdam Karl-Liebknecht-Str. 24–25 14476 Potsdam-Golm Germany; ^4^ Chair of Polymer Materials and Polymer Technologies Institute of Chemistry University of Potsdam Karl-Liebknecht-Str. 24–25 14476 Potsdam-Golm Germany

**Keywords:** galactose-decorated monomer, *glyco-inside* nano-assemblies, morphology evolution, PISA, RAFT dispersion polymerization

## Abstract

Glyco‐assemblies derived from amphiphilic sugar‐decorated block copolymers (ASBCs) have emerged prominently due to their wide application, for example, in biomedicine and as drug carriers. However, to efficiently construct these glyco‐assemblies is still a challenge. Herein, we report an efficient technology for the synthesis of glyco‐inside nano‐assemblies by utilizing RAFT polymerization of a galactose‐decorated methacrylate for polymerization‐induced self‐assembly (PISA). Using this approach, a series of highly ordered glyco‐inside nano‐assemblies containing intermediate morphologies were fabricated by adjusting the length of the hydrophobic glycoblock and the polymerization solids content. A specific morphology of complex vesicles was captured during the PISA process and the formation mechanism is explained by the morphology of its precursor and intermediate. Thus, this method establishes a powerful route to fabricate glyco‐assemblies with tunable morphologies and variable sizes, which is significant to enable the large‐scale fabrication and wide application of glyco‐assemblies.

## Graphical abstract

*Glyco‐inside* nano‐assemblies containing numerous intermediate morphologies are fabricated by leveraging RAFT polymerization of a galactose‐decorated monomer for polymerization‐induced self‐assembly. This method establishes a powerful route to fabricate *glyco‐inside* nano‐assemblies with tunable morphologies and variable sizes, which is significant to expand the large‐scale fabrication and wide application of glyco‐assemblies.

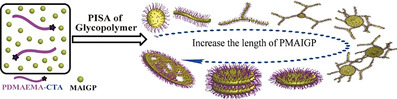

Glycopolymers, as one class of artificial polymers containing carbohydrates, have attracted great attention due to their roles in understanding and mimicking biological processes of carbohydrates including modulating intercellular communication, cell adhesion, and immunological properties.^[1]^ Glycopolymers made of amphiphilic sugar‐decorated block copolymers (ASBCs) are attractive because of the outstanding control over the chemical composition and conformation to achieve predefined chemical and physical properties.^[2]^ To utilize the excellent biocompatibility and unique biological performance of glycopolymers, various glyco‐assemblies based on ASBCs have been formed.^[3]^ Typically, glyco‐assemblies can be distinguished by the location of the sugars moieties in the aggregates: assemblies with sugars located on the outer layer of the nano‐objects can be assigned as *glyco‐outside* nano‐assemblies, while with sugars as the inner framework of the nanomaterials as *glyco‐inside* nano‐assemblies.^[4]^

Currently, main efforts in the research of glycopolymers in materials fields are utilizing the specific recognition performance of carbohydrates to develop smart glyco‐assemblies for drug carriers, bio‐sensors and biomedicine, among others.^[5]^ However, the multiple hydroxyl groups in monosaccharides influence the synthesis of related monomers and glycopolymers and further slow down the progress in the exploration of glyco‐assemblies. Moreover, to design smart glyco‐assemblies in which the biological function of the sugar is tunable is still a challenge. The protection‐deprotection strategy developed in glycochemistry provides a guidance for solving the above challenge through *glyco‐inside* nano‐assemblies. On the one hand, protected hydroxyl groups of sugars simplify the preparation procedure of sugar‐decorated monomers and lead to higher yields. On the other hand, the sugar function can be shielded and liberated by protecting and deprotecting the hydroxyl groups. Due to the great potential of *glyco‐inside* nano‐assemblies, numerous efforts have been undertaken to realize their fabrication and application.[^4^, ^6^] For instance, Jiang and Chen's group synthesized *glyco‐inside* nano‐assemblies with the morphology of micelles and vesicles.[^4^, ^6a^] Subsequently, they designed a smart carrier employing the *glyco‐inside* polymersome for a deprotection‐induced morphology transition, realizing an enzyme‐triggered release of an antigen.^[6b]^ Du's group developed a sugar‐breathed polymersome by incorporating glucosyl moieties in the inside of nano‐objects, realizing the regulation of the glucose level through the recognition interactions between ConA and glucose.^[7]^ Up to now, the classic approach to obtain *glyco‐inside* nano‐assemblies is selective‐solvent self‐assembly of ASBCs, in which sugars act as the hydrophobic moieties. This method has been developed for a long time and realized the fabrication of a series of glyco‐assemblies with different functions[^5c^, ^6b^, ^7^, ^8^] and various morphologies.^[9]^ However, complicated preparation procedures and low yields associated with this technique limit further application of ASBC‐based glyco‐assemblies.

Polymerization‐induced self‐assembly (PISA) is an excellent alternative to overcome those previously mentioned difficulties in the preparation of ASBC‐based glyco‐assemblies.^[10]^ PISA is an emerging technique realizing the fabrication of polymeric nano‐objects in suit during the synthesis of block polymers.^[11]^ Besides the simplified procedure, the polymeric nanoparticles are obtained by PISA in a high concentration and show a broad range of morphologies compared to the traditional selective‐solvent self‐assembly method. However, to the best of our knowledge, employing PISA to construct *glyco‐inside* nano‐assemblies has not been reported yet. The main challenge in synthesizing these *glyco‐inside* nano‐assemblies via PISA is finding a solvent system compatible with sugar‐monomers and resulting in a solvophobic glycopolymer to form the core layer of the nanostructures. It is well known that almost all sugar‐monomers and their homopolymers are hydrophilic, but protecting the hydroxyl groups of the carbohydrates leads to hydrophobic sugar‐monomers and polymers. In this case, it is difficult to utilize aqueous PISA to fabricate *glyco‐inside* nano‐assemblies. Fortunately, while employing a protection group strategy to obtain a galactose‐decorated methacrylate monomer, 6‐O‐methacryloyl‐1,2;3,4‐di‐O‐isopropylidene‐D‐galactopyranose (MAIGP), we found that MAIGP could be feasible for acting as the core‐forming monomer in an alcohol PISA system: MAIGP is soluble in methanol, but PMAIGP is insoluble in methanol.

Here, we demonstrate an efficient method for constructing highly ordered *glyco‐inside* nano‐assemblies by alcohol RAFT dispersion polymerization of the galactose‐decorated monomer MAIGP in the presence of poly((2‐dimethylamine)ethyl methacrylate)) (PDMAEMA) as macromolecular chain transfer agent (macro‐CTA, *M*_n_=4400 g mol^−1^, *M*_w_/*M*_n_=1.06, Figure S1). Methanol was chosen as the polymerization medium, as it is commonly used^[12]^ and fulfills the prerequisites for employing MAIGP as core‐forming monomer in the PISA system. By increasing PMAIGP length, spheres, linear worms, branched worms, highly branched worms, interwoven worms, lamellae, multilayer lamellae and complex vesicles were produced (Scheme 1).

**Scheme 1:**
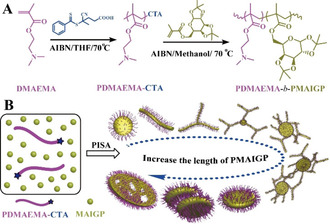
Synthesis of glyco‐nanostructures via RAFT dispersion polymerization (A). Illustration for the preparation of glyco‐nanostructures via PISA of PDMAEMA‐*b*‐PMAIGP, exhibiting a morphology transformation variation modulated by the length of the PMAIGP block (B).

In order to investigate the assembly behavior and constitutions of *glyco‐inside* nano‐assemblies obtained by PISA, a kinetic study of the RAFT dispersion polymerization of MAIGP was performed with ratio of AIBN/macro‐CTA/MAIGP=0.1/1/150 (see Table S1 for details). Photo images with laser irradiation were used to trace the change in appearance of the polymerization mixture after different reaction times, as shown in Figure 1 A and Figure S2. The appearance of the mixture changes from pellucid to bluish, and finally magnolia. Meanwhile, the size evolution of the formed assemblies was followed by dynamic light scattering (DLS) as shown in Figure 1 B and Figure S3. Furthermore, transmission electron microscopy (TEM) demonstrates changes in the morphology of the obtained glyco‐assemblies (Figure 1 A). Up until 3 h reaction time, the size of the mixture stays at approximately 4 nm and no aggregation is visible (Figure S4). The reason why the DLS results until 4 h reaction time are larger than 0 nm (Table S1) might be the inherent hydrodynamic volume of PDMAEMA chains in highly concentrated solution. Prolonging polymerization time from 4 h to 24 h, the particle sizes increased from 47 nm to 1.0 μm accompanied with the morphology variation from spherical micelles to multilayer lamellae.

**Figure 1:**
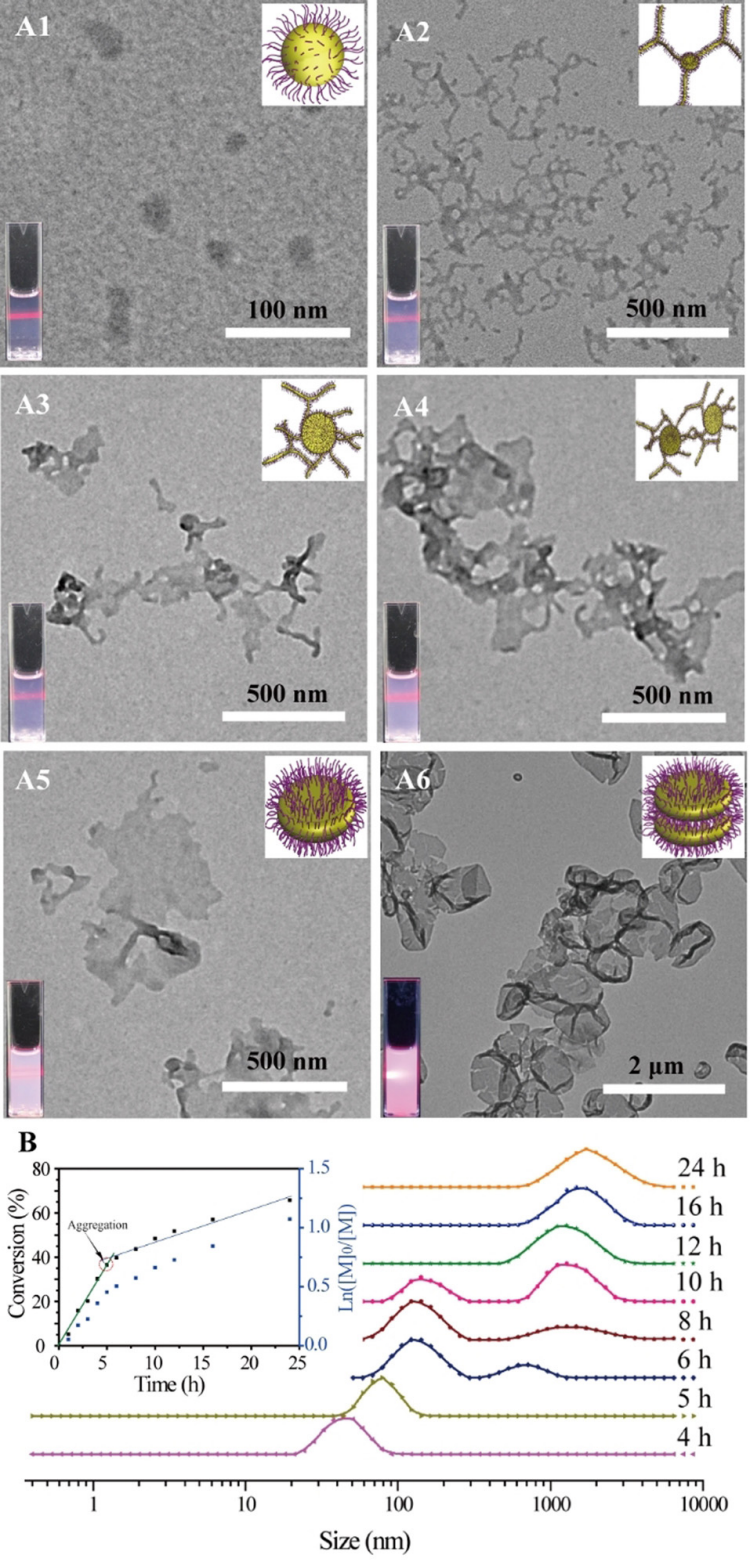
TEM and photo images (laser irradiation) of the mixture after periodical polymerization times (A), A1: 4 h, A2: 6 h, A3: 8 h, A4: 10 h, A5: 12 h, A6: 24 h. Normalized DLS results of the obtained mixture after periodical polymerization times (B). Insert: MAIGP conversion with polymerization time. RAFT dispersion polymerization of MAIGP in methanol at 70 °C using PDMAEMA_28_ as macro‐agent; [MAIGP]/[PDMAEMA]/[AIBN]=150:1:0.1, solids content=5 %.

To further investigate the assembly behavior of glyco‐assemblies in terms of copolymer constitution, the monomer conversions were followed by ^1^H‐NMR spectroscopy (Figure S5). From the polymerization time‐conversion curves (Figure 1 B insert), it can be concluded that the polymerization undergoes two stages: the first stage occurs within 5 h polymerization time, corresponding to the solution polymerization of MAIGP. The rate decreases after 5 h, which corresponds to the decreased concentration of monomer within the nanoparticles after micelles formed in the reaction mixture and limited transfer of monomer from the solution to the particles. These results correspond with the TEM images. Only about 70 % monomer was consumed after 24 h, probably because of the low reactivity of MAIGP resulting from steric hindrance due to the multi‐ring structure in the MAIGP monomer. We used size‐exclusion chromatography (SEC) to study the characteristics of the obtained glycopolymers, as shown in Figure S6. The *M*_n_ of the copolymers increases linearly with prolonged polymerization times, and the polydispersity index (*M*_w_/*M*_n_) stays narrow even after high monomer conversion (Figure S7). These results suggest that employing the galactose‐decorated monomer MAIGP as the core‐forming monomer could realize the fabrication of *glyco‐inside* nano‐assemblies via PISA.

According to previous research of PISA, the morphologies of nano‐objects obtained via PISA are influenced by many parameters, including the length of hydrophilic and hydrophobic segments, the solids content during polymerization, the solvent, etc.^[13]^ To determine the parameters for constructing *glyco‐inside* nano‐assemblies via PISA, a series of RAFT dispersion polymerizations were carried out by varying the targeted DP of MAIGP (DP_PMAIGP_) from 40 to 150 and the solids content from 5 % to 20 %. Polymerization details can be found in Table S2. The achieved nano‐objects are named as PDMAEMA_28_‐PMAIGP_*n*_‐X (n corresponds to the DP of PMAIGP, and X stands for the solids content of the polymerization mixture). The monomer conversions and constitutions of PDMAEMA_28_‐PMAIGP_n_‐X were determined by ^1^H‐NMR spectroscopy and SEC. ^1^H‐NMR spectra (Figure S8–S11) and SEC curves (Figure S12–S15) of the obtained glycopolymers PDMAEMA_28_‐PMAIGP_*n*_‐X demonstrate that the DPs of PMAIGP are close to the targeted DP and the molecular weight distributions are relatively narrow (*M*_w_/*M*_n_≤1.2).

The morphologies and sizes of the obtained glyco‐assemblies under different DP_PMAIGP_ and solids contents were characterized by TEM (Figure S16–S19) and DLS (Figure S20–S23). When the RAFT dispersion polymerization was performed at 5 % solids content, the morphologies varied from spherical micelles to lamella, containing many intermediate morphologies such as linear worms, branched worms, highly branched worms, jellyfishes, lamellae and multilayer lamellae (as shown in Figure S16). Meanwhile, the DLS results (Figure S20) show a gradually varying size from 40 nm to 1.0 μm. When we increased the solids content to 10 %, another morphology besides the previously mentioned ones appeared, namely crossed jellyfish (Figure S17). When further increasing the solids content to 15 %, many of the intermediate morphologies disappeared, but an advanced aggregation of complex vesicles was found (Figure S18). When the solids content increased to 20 %, we could observe complex vesicles if the PMAIGP chain was extended enough (Figure S19). To confirm the morphologies we observed, scanning electron microscopy (SEM) measurements were employed. The SEM images (Figure S24 and Figure S25) show similar structures as the TEM investigations. Subsequently, to exclude the influence of sample preparation on the morphologies (especially solvent evaporation), we selected representative samples (D_28_M_40_‐5 % and D_28_M_50_‐10 %) to perform cryogenic transmission electron microscopy (cryo‐TEM). Therefore, the samples were diluted with water and their cryo‐TEM images show nearly unaltered structures compared to the TEM and SEM images (Figure S26 and Figure S27). The DLS results show that the size change of these glyco‐assemblies is accompanied with the morphology evolution. From these results, it becomes obvious that various highly ordered *glyco‐inside* nano‐assemblies could be fabricated by either adjusting the DP_PMAIGP_ or the polymerization solids content. The DP of the PMAIGP chain and the solids content were systematically varied to construct a phase diagram that enables the reproducible preparation of morphologically pure glyco‐assemblies (Figure 2 A). Representative morphologies for various *glyco‐inside* nano‐assemblies are shown in Figure 2 B. Notably, the phase diagram presents a correlation between solids content or DP_PMAIGP_ and morphology, providing a guidance to select the optimal parameter for fabricating *glyco‐inside* nano‐assemblies with specific and pure morphology. The morphology evolution process induced by the length of PMAIGP and solids content is similar to published theory simulation results based on self‐consistent field theory (SCFT).^[14]^

**Figure 2:**
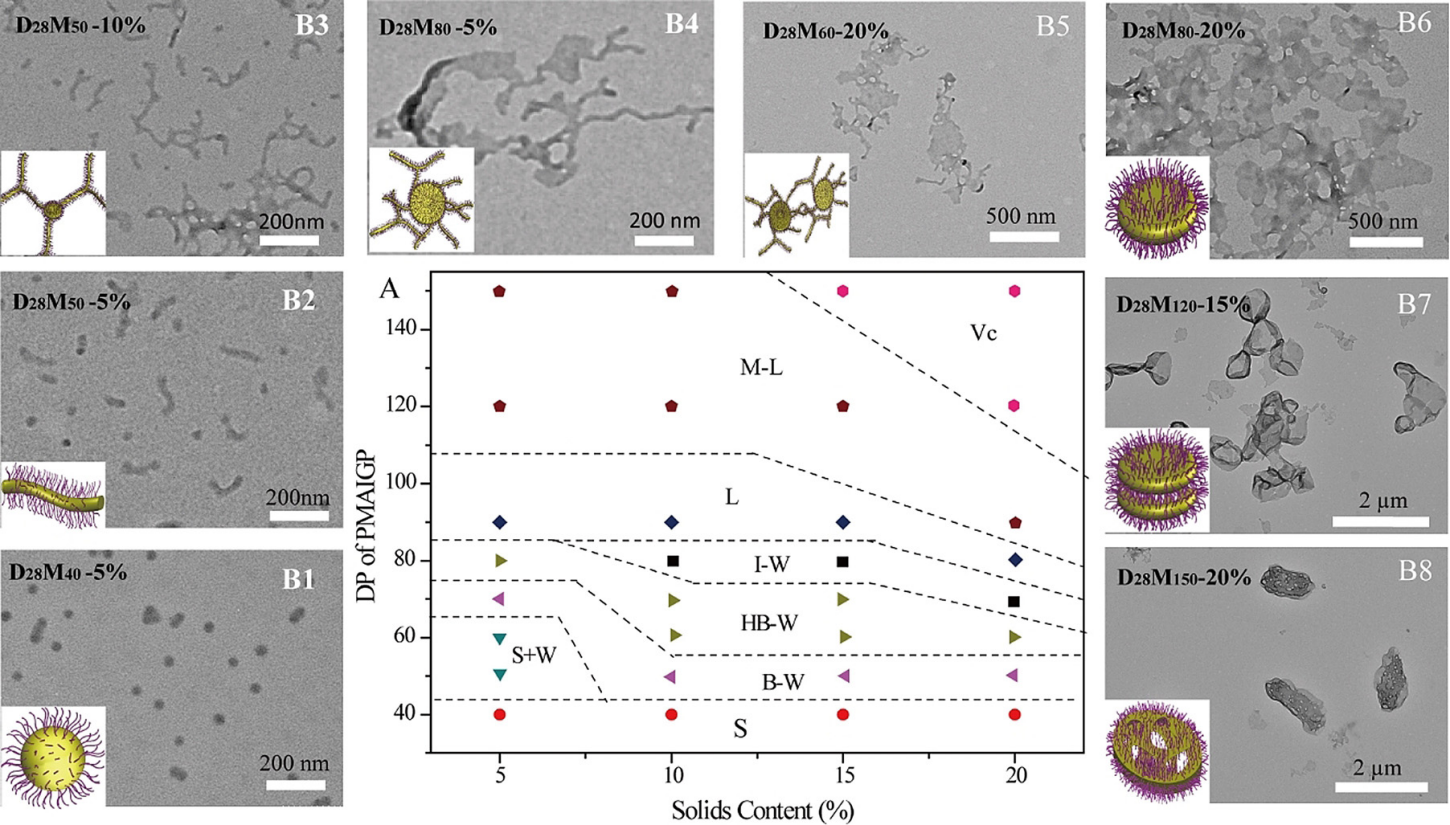
Phase diagram (A) derived for glyco‐assemblies of PDMAEMA_28_‐PMAIGP_*n*_‐X (D_28_M_*n*_‐X) obtained by RAFT dispersion polymerization of MAIGP in methanol at 70 °C. TEM images of representative morphologies of glyco‐assemblies: B1 spheres, B2 mixture of spheres and linear worms, B3 branched worms, B4 highly branched worms, B5 interwoven worms, B6 lamellae, B7 multilayer lamellae, B8 complex vesicles.

Of particular interest for us was the unusual morphology of complex vesicles (Figure 3 A and Figure S25) observed when adjusting the DP_PMAIGP_ under high solids content (PDMAEMA_28_‐PMAIGP_150_‐20 %) and no vesicular structure precursor appeared during the synthesis process. According to the previous research, complex vesicles^[13c]^ are an advanced chain aggregation state compared to vesicles.^[15]^ Until now, no study reports the fabrication of glyco‐assemblies with complex vesicle morphology, neither via traditional method nor via PISA approach. To understand the formation process of the complex vesicle glyco‐structures during RAFT dispersion polymerization of MAIGP, the resulting glyco‐assemblies at solids content of 20 % were further investigated. As shown in Figure 3 and Figure S19, when increasing the DP_PMAIGP_ from 80 to 90, we found morphologies ranging from lamellae (Figure 3B1 and 3B2) to multilayer lamellae (Figure 3C1 and 3C2). For continued increase of DP_PMAIGP_ to 120 or even higher, complex vesicles (Figure 3D1 and 3D2) were observed. Thus, we predict the formation of complex vesicles during the PISA process (Figure 3 E): in terms of traditional PISA process, increasing solvophobic chain length, lamellae grow to form multilayer lamellae, and further evolve to vesicles in traditional PISA process. However, due to hydrogen‐bond interactions between PMAIGP chains and the surrounding medium (methanol), longer PMAIGP chains result in stronger inter‐chain interactions, which hinder the movement of PMAIGP chains. In this case, lamellae can only extend and fuse to form the observed multilayer lamellae. These multilayer lamellae grow and evolve to form imperfect vesicular structures of complex vesicles.

**Figure 3:**
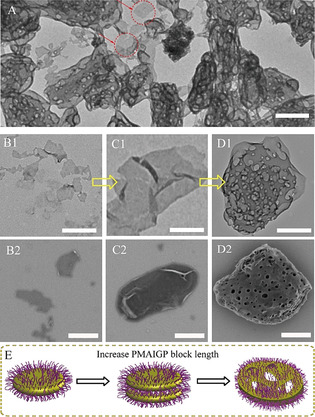
The morphology evolution process of glyco‐assemblies from lamellae to complex vesicles obtained via PISA of PDMAEMA‐PMAIGP. TEM images of representative intermediate structures: A) overview of complex vesicles. B1) lamellae, C1) multilayer lamellae, D1) detail of complex vesicles. SEM images of representative intermediate structures: B2) lamellae, C2) multilayer lamellae, D2) detail of complex vesicles. E) illustration of the evolution process in obtaining complex vesicles glyco‐assemblies. (scale bar=500 nm).

In conclusion, we realized the construction of highly ordered *glyco‐inside* nano‐assemblies by utilizing RAFT polymerization of a galactose‐decorated monomer for polymerization‐induced self‐assembly for the first time. A series of intermediate morphologies, including branched worms, highly branched worms, multilayer lamellae and complex vesicles were obtained by regulating either the length of the PMAIGP block or the solids content. The occurrence of these morphologies is attributed to the multi‐ring structure of PMAIGP and strong hydrogen bonding interactions between PMAIGP chains, which hinder the movement ability of PMAIGP during the chain‐extension process. The critical intermediate morphology of multilayer lamellae reveals the formation mechanism of complex vesicles: the lamellae fuse to form multilayer lamellae that grow to form imperfect vesicular structures. This approach provides guidance to large‐scale synthesis of highly ordered *glyco‐inside* nano‐assemblies with tuneable morphologies and variable sizes. With the current achievements in PISA of amphiphilic sugar‐decorated block copolymers, we envisage that the combination of PISA with glycopolymers will have a significant impact on glycobiology and nanoscience.

## Conflict of interest

The authors declare no conflict of interest.

## Supporting information

As a service to our authors and readers, this journal provides supporting information supplied by the authors. Such materials are peer reviewed and may be re‐organized for online delivery, but are not copy‐edited or typeset. Technical support issues arising from supporting information (other than missing files) should be addressed to the authors.

Supplementary
