# Supplementary material for: Construction of Highly Ordered Glyco‐Inside Nano‐Assemblies through RAFT Dispersion Polymerization of Galactose‐Decorated Monomer
Source: Angew Chem Int Ed Engl. 2021 Mar 25;60(20):11098–103. doi: 10.1002/anie.202015692 (PMC8252037; doi:10.1002/anie.202015692)
Supplement: Supplementary file 1 — Supplementary [file ANIE-60-11098-s001.pdf]

Angewandte Chemie
Eine Zeitschrift der Gesellschaft Deutscher Chemiker

Supporting Information

Construction of Highly Ordered Glyco-Inside Nano-Assemblies through RAFT Dispersion Polymerization of Galactose-Decorated Monomer

Liang Qiu, Haoran Zhang, Thomas Bick, Johannes Martin, Petra Wendler, Alexander Böker, Ulrich Glebe,* and Chengfen Xing**

anie_202015692_sm_miscellaneous_information.pdf

Content

1. Materials. 1

2. Characterizations.. 1

3. Synthesis... 2

3.1 Synthesis of PDMAEMA macro chain transfer agent (Macro-CTA)........................... 2

3.2 Synthesis of glycopolymeric nanoparticles of PDMAEMA-*b*-PMAIGP in methanol by RAFT dispersion polymerization. 2

3.3 Kinetics of Dispersion Polymerization of MAIGP with Targeting DP of 150. 2

4. The characteristics of macro chain transfer agent (Macro-CTA). 3

5. The investigation of polymerization dynamic study for RAFT dispersion polymerization of MAIGP 3

6. The investigation of polymerization parameter on the influence of *glyco-inside* nano-assemblies. 8

1. Materials.

Diacetone-D-galactose, 2-(dimethylamino)ethyl methacrylate (DMAEMA) and methacryloyl chloride were purchased from Aladdin. 2,2`-Azobis(2-methylpropionitrile) (AIBN) was purchased from Sinopharm Chemical Reagent co., ltd. and was recrystallized from methanol. DMAEMA was passed through alkaline alumina column before use. 6-O-Methacryloyl-1,2; 3,4-di-O-isopropylidene-D-galactopyranose (MAIGP)[1], 2-(2-hydroxyethoxy)ethyl methacrylate (HEO_2MA)[2] and 4-cyano-pentanoicacid dithiobenzoate (CPADB)[3] were prepared according to our previous reports. All other reagents were used as received.

2. Characterizations.

Nuclear Magnetic Resonance (NMR) spectroscopy. The ^{1}H-NMR (400 MHz) measurements were performed on Bruker DMX-400 spectrometer in $CDCl_3$ or DMSO-d_6.

Size-Exclusion Chromatography (SEC) Measurements. The molecular weight and molecular weight distribution of the PDMAEMA and PDMAEMA-*b*-PMAIGPn were determined on a Waters 150C gel permeation chromatography (GPC) equipped with three Ultrastyragel columns in series and RI 2414 detector at 30 °C, and THF was used as eluent at a flow rate of 1.0 mL/min. Monodispersed polystyrene standards were used for the calibration of molecular weight and molecular weight distribution.

Transmission Electron Microscope (TEM). TEM observations were performed on a Hitachi H-7700 TEM at an accelerating voltage of 110 kV. The samples were prepared by depositing a drop of the polymer solution in methanol on copper grids, and then allowed to dry at room temperature overnight.

Scanning electron microscope (SEM). SEM images were acquired on a FEI Nova Nano SEM 450. The samples for SEM measurements were prepared by placing a drop of the nanoparticle solution in methanol on silicon wafers and then allowed to dry at room temperature overnight.

Dynamic Light Scattering (DLS). A Malvern Z90 Zetasizer equipped with a 633 nm He−Ne laser and an avalanche photodiode detector was used to characterize the hydrodynamic size of the self-assemblies. The scattering light at 90° angle was detected and used to analyze the size and distribution. The concentration of *glyco-inside* nano-assemblies for DLS measurements was in the range of 1-5 mg/mL.

Cryogenic transmission electron microscopy (cryo-TEM). Cryo-TEM analysis was done on a Thermo Fisher Talos F200C G2 field emission gun TEM operating at 200 keV. The methanol samples were diluted with water to 5 mg/mL to a methanol content of only 2% v/v. For vitrification, 4 μL of the nano-assembly solution was applied to freshly glow discharged Quantifoil R3/3 300-mesh holey carbon grids with 2 nm carbon support film. The sample was incubated for 45 sec at 10°C and 90% humidity before blotting for 3 sec and plunge freezing into liquid ethane using the sensor blotting function on a Leica EM GP2 plunge freezer. Images were taken on a 4k x 4k Ceta 16M CMOS camera.

3. Synthesis.

3.1 Synthesis of PDMAEMA macro chain transfer agent (Macro-CTA)

A typical protocol for the synthesis of a $PDMAEMA_{28}$ macro-CTA was as follows: A solution of DMAEMA (4680 mg, 30 mmol), CPADB (208 mg, 0.75 mmol), AIBN (12.3 mg, 0.075 mmol), in 4.9 mL of ethanol was added into a 10 mL tube. The tube was sealed under vacuum after freeze–pump–thaw cycles, and then immersed in an oil bath at 70 °C for 6 h. The polymerization was quenched by immersing the tube in ice water. The PDMAEMA was isolated by precipitation in n-hexane followed by filtration and then dried under vacuum overnight. The final conversion (70%) and DP (28) were determined by ^{1}H-NMR spectroscopy. SEC analysis indicated M_n and M_w/M_n values as 2900 g mol^{-1} and 1.06, respectively.

3.2 Synthesis of glycopolymeric nanoparticles of PDMAEMA-*b*-PMAIGP in methanol by RAFT dispersion polymerization.

A series of RAFT dispersion polymerizations with feed molar ratios of MAIGP/$PDMAEBA_{40}$ ranging from 30 to 150 and $PDMAEMA_{40}$/AIBN = 1/0.2 at solid content of 5 wt %, 10 wt %, 15 wt %, or 20 wt % in methanol were carried out at 70 °C for 24 h. A typical procedure is described as follows: $PDMAEMA_{28}$ macro-CTA (22 mg, 5 μmol), AIBN (0.164 mg, 1 μmol, CTA/AIBN molar ratio = 5/1), and MAIGP (246 mg, 0.75 mmol) were dissolved in methanol (5.092 g, 5 w/w %), and then the solution was added into a 10 mL glass tube. The tube was sealed after three freeze–pump–thaw cycles, and then placed in a preheated oil bath at 70 °C for 24 h. The polymerization was stopped by rapidly immersing the tube in ice water. A drop of the reaction mixture was taken for ^{1}H-NMR measurement, and the MAIGP conversion was calculated based on the integral values of the methine (-O-CH-O-) proton signal at 5.05 ppm and the ester methylene (-CH_2-O-C(O)-) proton signal at 3.8–4.5 ppm. To measure ^{1}H-NMR spectra and SEC curves of the diblock copolymers, $PDMAEMA_{28}$-*b*-PMAIGP, a small amount of the reaction mixture was diluted with THF, and the diluted solution was dropped into excess n-hexane while stirring; then the precipitate was obtained by filtration. The solid was dried in a vacuum oven at room temperature overnight. For TEM and DLS measurements, a small portion of the reaction mixture was diluted with methanol, and the TEM and SEM sample was prepared by depositing a drop of the diluted dispersion onto a copper grid or silicon wafers, subsequently drying at room temperature.

3.3 Kinetics of Dispersion Polymerization of MAIGP with Targeting DP of 150.

$PDMAEMA_{28}$ macro-CTA (107.5 mg, 0.025 mmol), MAIGP (1.23 g, 3.75 mmol), 4-hydroxybenzaldehyde (HBA) (0.457 g, 3.75 mmol, MAIGP/HBA molar ratio = 1:1) and AIBN (0.82 mg, 0.005 mmol, CTA/AIBN molar ratio = 10:1) were dissolved in methanol (8.5 mL, 5 w/w %). The mixture was evenly divided into 10 tubes and these tubes were sealed after three freeze–pump–thaw cycles. One tube was immediately taken for ^{1}H-NMR analysis as the "zero time" (t = 0 min) for this polymerization. Others were placed in a preheated oil bath at 70 °C and terminated at various time intervals for ^{1}H-NMR and TEM analysis. ^{1}H-NMR samples were quenched by dilution in DMSO-d_6 at 20 °C. Monomer conversions were normalized using the MAIGP monomer as an internal standard, and are expressed relative to the ratio of the signals at ζ=6.1 ppm and ζ=5.5 ppm observed at "zero time". For TEM analysis, the sample was prepared by depositing a drop of the diluted dispersion onto a copper grid, followed by drying at room temperature.

4. The characteristics of macro chain transfer agent (Macro-CTA).

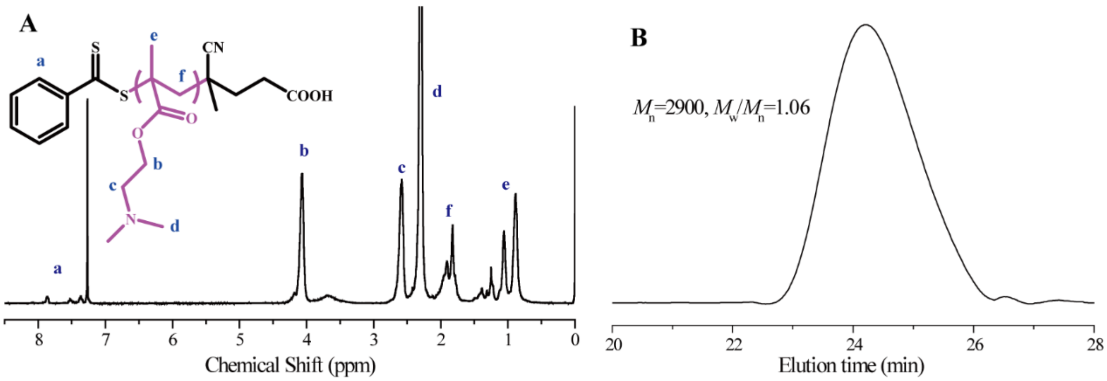

Figure S1. ^{1}H-NMR spectrum (A) and SEC curve (B) of the macro chain transfer agent (macro-CTA) of $P(DMAEMA)_{28}$.

5. The investigation of polymerization dynamic study for RAFT dispersion polymerization of MAIGP

Table S1. Overview of the polymerization dynamic study of RAFT dispersion polymerization of MAIGP[a]

Time (h)	Conv.[b] (%)	*DP*[b]	M_n[c] kg/mol	M_w/M_n[c]	Size[d] (nm)	Morph.[e]
1	5.16	8	4000	1.06	2.6	-
2	15.83	24	5300	1.08	3.2	-
3	20.19	30	6400	1.07	4.1	-
4	30.25	45	8000	1.09	47.0	S
5	36.54	55	9100	1.07	74.0	S
6	39.71	60	10200	1.03	-	L-W & B-W
8	43.68	66	10700	1.03	-	HB-W
10	48.45	73	11200	1.03	-	I-W
12	51.74	78	12800	1.04	800	L
16	57.01	86	15900	1.05	1000	L
24	65.78	99	16100	1.08	1200	M-L

[a] The polymerization was performed in methanol at 70 °C at solids content of 5 % and with feed ratio of AIBN/PDMAEMA/MAIGP=0.1/1/150. [b] Conversion of MAIGP and DP determined by ^{1}H-NMR analysis. [c] M_n and M_w/M_n measured by SEC. [d] Size determined by DLS. [e] Morph. refers to morphology, determined by TEM. S (spheres), L-W (linear worms), B-W (branched worms), HB-W (highly branched worms), I-W (interwoven worms), L (lamellae), M-L (multilayer lamellae), Vc (complex vesicles).

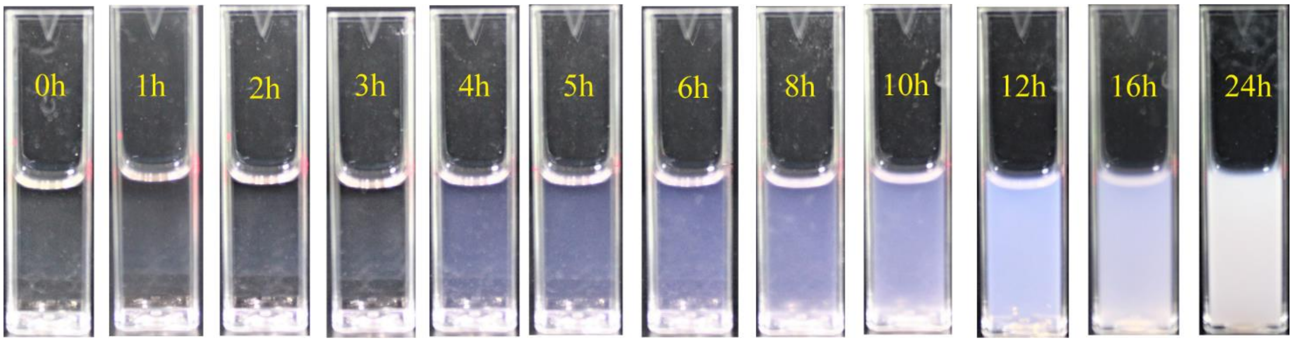

Figure S2. Photos of the reaction mixture after scheduled polymerization time. The polymerization was performed in methanol at 70 °C, at solids content of 5%, with the feed ratio of AIBN/PDMAEMA/MAIGP=0.1/1/150, and $PDMAEMA_{28}$ as the macro-CTA.

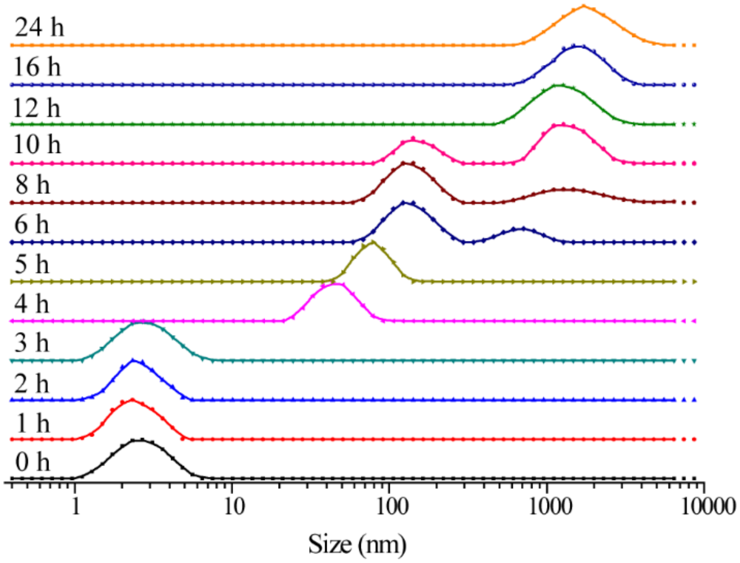

Figure S3. Normalized DLS curves of $PDMAEMA_{28}$-PMAIGP after scheduled polymerization time. The polymerization was performed in methanol at 70 °C, at solids content of 5%, with the feed ratio of AIBN/PDMAEMA/MAIGP=0.1/1/150, and $PDMAEMA_{28}$ as the macro-CTA.

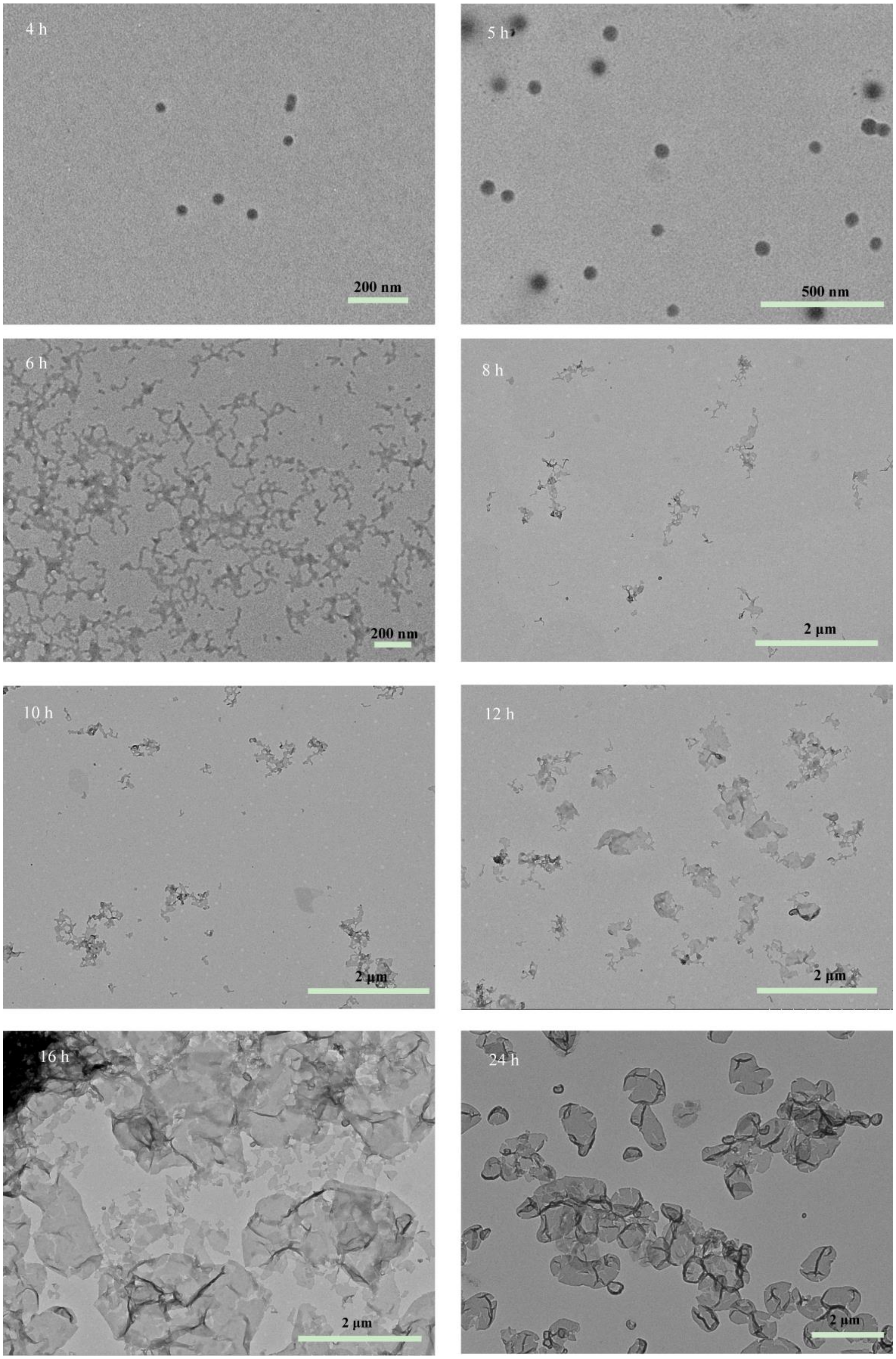

Figure S4. TEM images of $PDMAEMA_{28}$-PMAIGP after scheduled polymerization time. The polymerization was performed in methanol at 70 °C, at solids content of 5%, with the feed ratio of AIBN/PDMAEMA/MAIGP=0.1/1/150, and PDMAEMA as the macro-CTA.

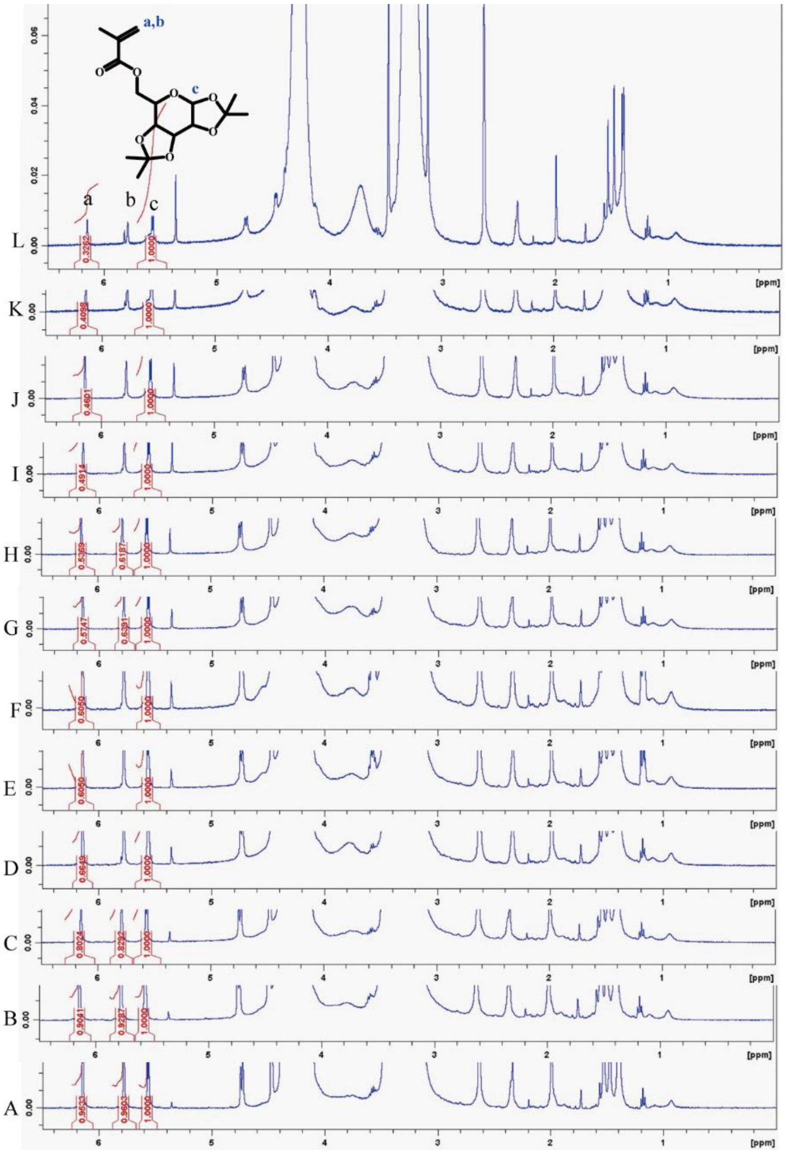

Figure S5. ^{1}H-NMR spectra of the reaction media after polymerization for (A) 0 h, (B) 1 h, (C) 2 h, (D) 3 h, (E) 4 h, (F) 5 h, (G) 6 h, (H) 8 h, (I) 10 h, (J) 12 h, (K) 16 h and (L) 24 h. 150 µL of the reaction mixture was taken for ^{1}H-NMR spectroscopy (DMSO-d_6) at scheduled time of polymerization. The polymerization was performed in methanol at 70 °C, at solids content of 5%, with the feed ratio of AIBN/PDMAEMA/MAIGP=0.1/1/150, and PDMAEMA as the macro-CTA.

The conversions of MAIGP were calculated according to the following equation:

Conversion $(\%)_{MAIGP} = (1 - a_t/a_0) \times 100\%$

a_0 is the integral value of the proton in MAIGP (signal a) at 0 h of polymerization. a_t is the integral value of the proton in MAIGP (signal a) at t h of polymerization.

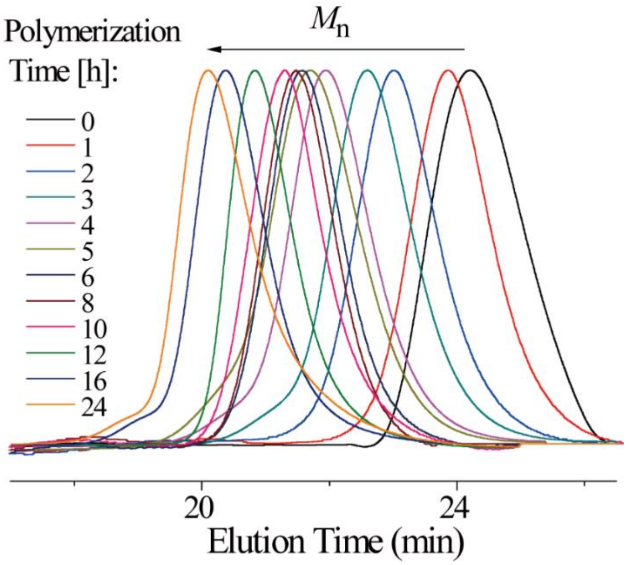

Figure S6. The evolution of SEC traces of PDMAEMA$_{28}$-*b*-PMAIGP$_x$ after scheduled polymerization time. The polymerization was performed in methanol at 70 °C, at solids content of 5%, with the feed ratio of AIBN/PDMAEMA/MAIGP=0.1/1/150, and PDMAEMA as the macro-CTA.

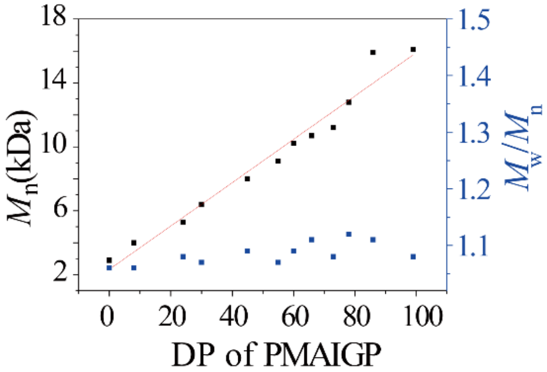

Figure S7. The curve of PDMAEMA$_{28}$-b-PMIGP$_x$ molecular weight (measured by SEC) and polydispersity index (determined by SEC) vs DP of PMAIGP. RAFT dispersion polymerization of MAIGP was performed in methanol using PDMAEMA$_{28}$ as macro-agent at 70 °C, [MAIGP]/[PDMA]/[AIBN] = 150:1:0.1, solids content = 5%.

6. The investigation of polymerization parameter on the influence of *glyco-inside* nano-assemblies.

Table S2. Synthesis parameters and characterization of PDMAEMA-*b*-PMAIGP nano-objects prepared by RAFT dispersion polymerization in methanol at 70 °C[a].

Solid Content(%)	Target DP[a] (MAIGP)	Actual DP[b] (MAIGP)	Con[c] (%)	$M_{n(NMR)}$[c] kg/mol	$M_{n(SEC)}$[d] kg/mol	$M_w/M_{n(SEC)}$[d]	Morp.[e]	DLS[f] (nm)	PDI[f]
5	40	40	100	17400	9400	1.10	S	48	0.125
	50	50	100	20700	10700	1.09	S+W	-	-
	60	60	100	24000	12200	1.09	S+W	-	-
	70	64	91.4	25300	13060	1.10	B-W	-	-
	80	77	96.2	29600	13400	1.09	HB-W	-	-
	90	76	84.4	29200	14100	1.09	L	707	0.341
	120	109	90.8	40100	18000	1.11	M-L	1039	0.134
	150	134	89.3	48300	23000	1.14	M-L	1577	0.279
10	40	40	100	17400	10100	1.07	S	39	0.149
	50	50	100	20700	12500	1.08	B-W	-	-
	60	60	100	24000	13400	1.08	HB-W	-	-
	70	65	92.8	25600	14700	1.09	HB-W	-	-
	80	78	97.5	29900	15100	1.09	I-W	-	-
	90	83	92.2	31500	16100	1.11	L	750	0.454
	120	110	91.7	40400	18900	1.14	M-L	1863	0.612
	150	131	87.3	47300	24869	1.18	M-L	1041	0.421
15	40	40	100	17400	10400	1.08	S	36	0.197
	50	50	100	20700	11400	1.08	B-W	-	-
	60	60	100	24000	13500	1.09	HB-W	-	-
	70	65	93	25600	14400	1.09	HB-W	-	-
	80	80	100	30500	16300	1.10	I-W	-	-
	90	90	100	33800	18800	1.12	L	523	0.29
	120	110	91.7	40400	21000	1.13	M-L	656	0.38
	150	139	93	49900	25900	1.17	Vc	1002	0.26
20	40	40	100	17400	10600	1.10	S	35	0.220
	50	50	100	20700	12400	1.09	B-W	-	-
	60	60	100	24000	13600	1.10	HB-W	-	-
	70	70	100	27300	15300	1.09	I-W	-	-
	80	80	100	30500	17400	1.12	L	670	0.225
	90	90	100	33800	19400	1.11	M-L	629	0.413
	120	111	93	40700	23400	1.15	Vc	1629	0.317
	150	137	91	49200	30900	1.20	Vc	1468	0.252

[a] The polymerization with different target degree of polymerization of MAIGP and solids content was performed in methanol at 70 °C for 24 h, $PDMAEMA_{28}$ as macro-agent at 70 °C, [PDMAEMA]/[AIBN] = 1:0.2.
[b] Determined by ^{1}H-NMR data.
[c] Calculated from ^{1}H-NMR data.
[d] Measured by SEC.
[e] Morp. refers to morphology, determined from TEM images. S, L-W, B-W, HB-W, I-W, L, M-L, stand for spheres, linear worms, branched worms, highly branched worms, interwoven worms, lamellas and multilayer lamellas, respectively
[f] Size refers to the diameter of these nano-objects, which was calculated by dynamic light scattering.

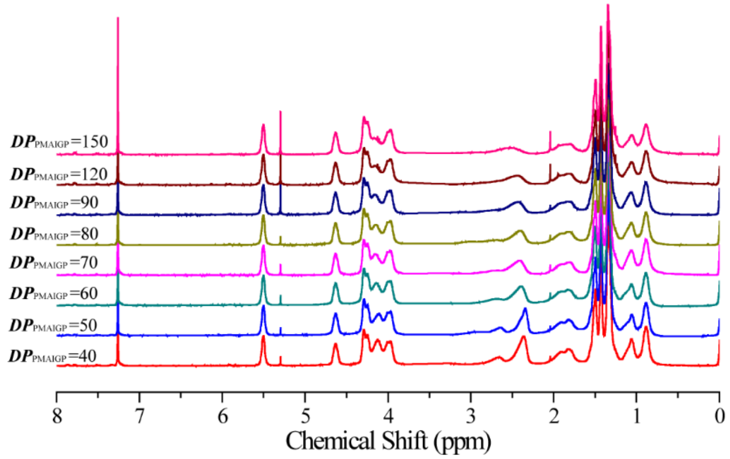

Figure S8. ^{1}H-NMR spectra of $PDMAEMA_{28}$-$PMAIGP_n$ nano-objects with various target degree of polymerization of MAIGP at solids content of 5%, $PDMAEMA_{28}$ as macro-agent at 70 °C, [PDMAEMA]/[AIBN] = 1:0.2.

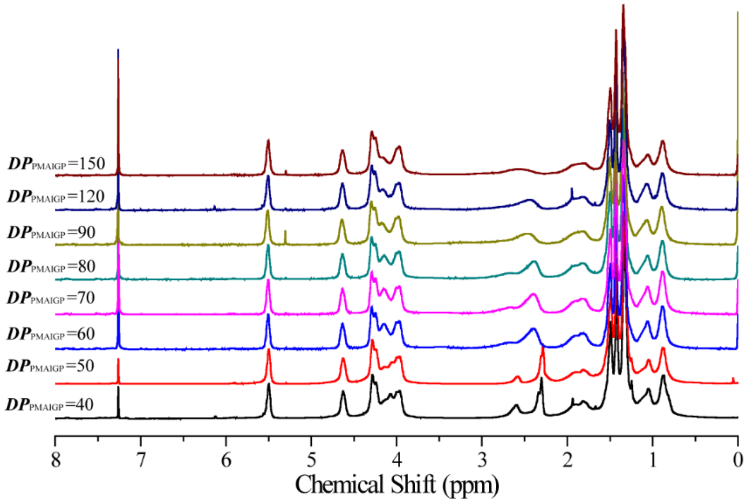

Figure S9. ^{1}H-NMR spectra of $PDMAEMA_{28}$-$PMAIGP_n$ nano-objects with various target degree of polymerization of MAIGP at solids content of 10%, $PDMAEMA_{28}$ as macro-agent at 70 °C, [PDMAEMA]/[AIBN] = 1:0.2.

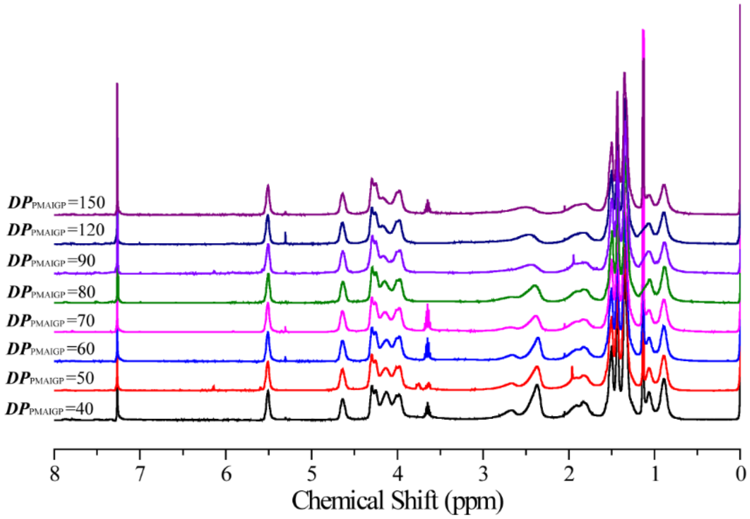

Figure S10. ^{1}H-NMR spectra of $PDMAEMA_{28}$-$PMAIGP_n$ nano-objects with various target degree of polymerization of MAIGP at solids content of 15%, $PDMAEMA_{28}$ as macro-agent at 70 °C, [PDMAEMA]/[AIBN] = 1:0.2.

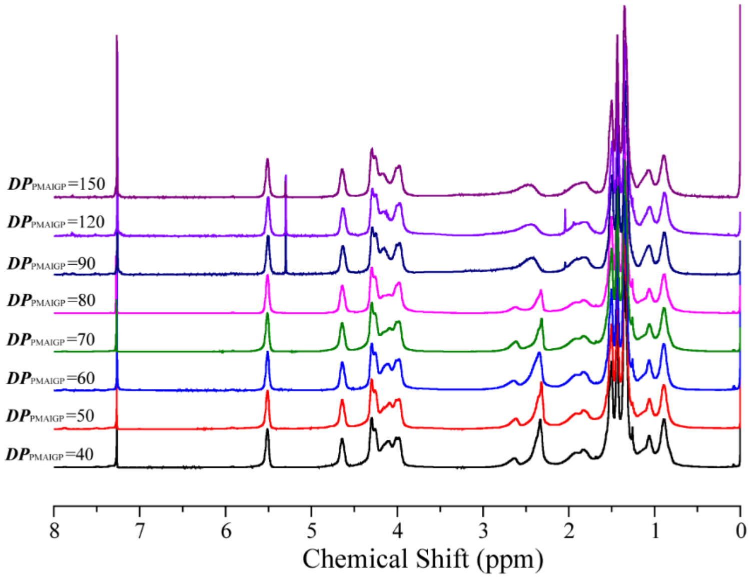

Figure S11. **^{1}H-NMR spectra of PDMAEMA$_{28}$-PMAIGP$_n$ nano-objects with various target degree of polymerization of MAIGP at solids content of 20%, PDMAEMA$_{28}$ as macro-agent at 70 °C, [PDMAEMA]/[AIBN] = 1:0.2.**

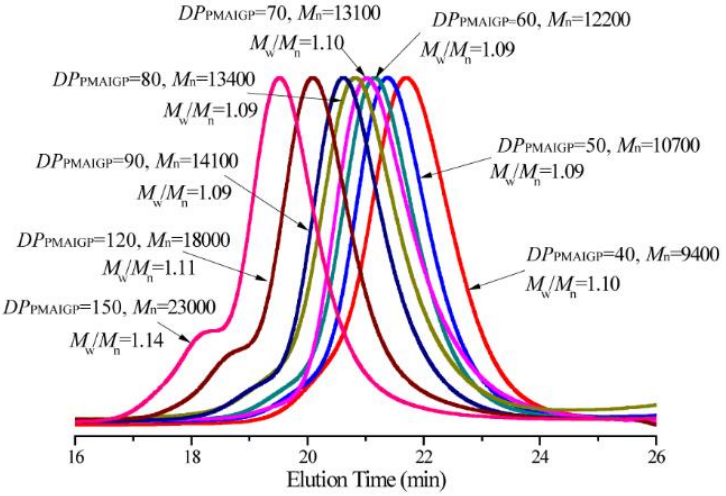

Figure S12. SEC traces of PDMAEMA$_{28}$-PMAIGP$_x$ nano-objects with various target degree of polymerization of MAIGP at solids content of 5%, PDMAEMA$_{28}$ as macro-agent at 70 °C, [PDMAEMA]/[AIBN] = 1:0.2.

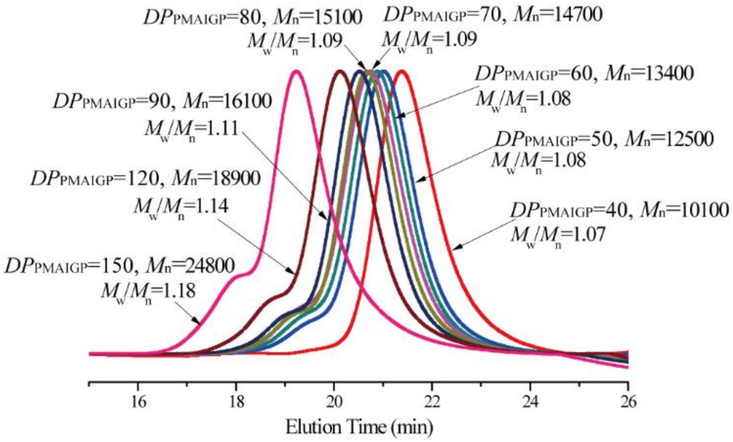

Figure S13. SEC traces of $PDMAEMA_{28}$-$PMAIGP_x$ nano-objects with various target degree of polymerization of MAIGP performed at solids content of 10%, $PDMAEMA_{28}$ as macro-agent at 70 °C, [PDMAEMA]/[AIBN] = 1:0.2.

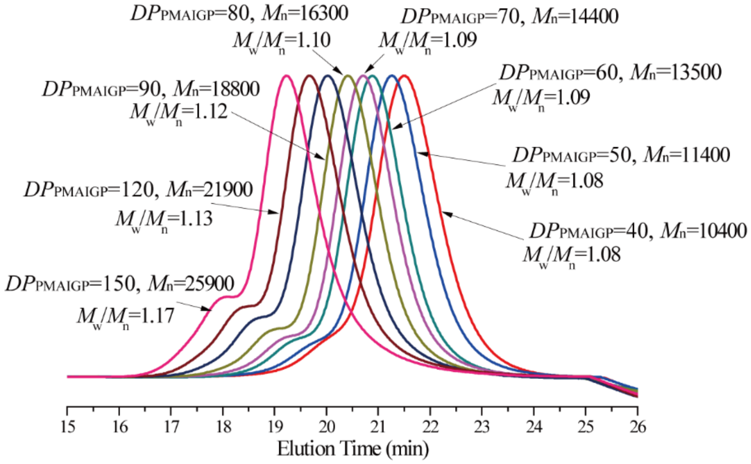

Figure S14. SEC traces of $PDMAEMA_{28}$-$PMAIGP_x$ nano-objects with various target degree of polymerization of MAIGP at solids content of 15%, $PDMAEMA_{28}$ as macro-agent at 70 °C, [PDMAEMA]/[AIBN] = 1:0.2.

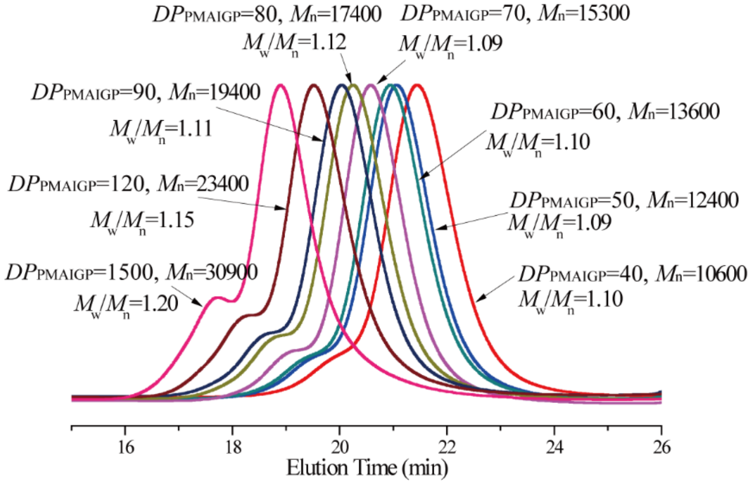

Figure S15. SEC traces of $PDMAEMA_{28}$-$PMAIGP_x$ nano-objects with various target degree of polymerization of MAIGP at solids content of 20%, $PDMAEMA_{28}$ as macro-agent at 70 °C, [PDMAEMA]/[AIBN] = 1:0.2.

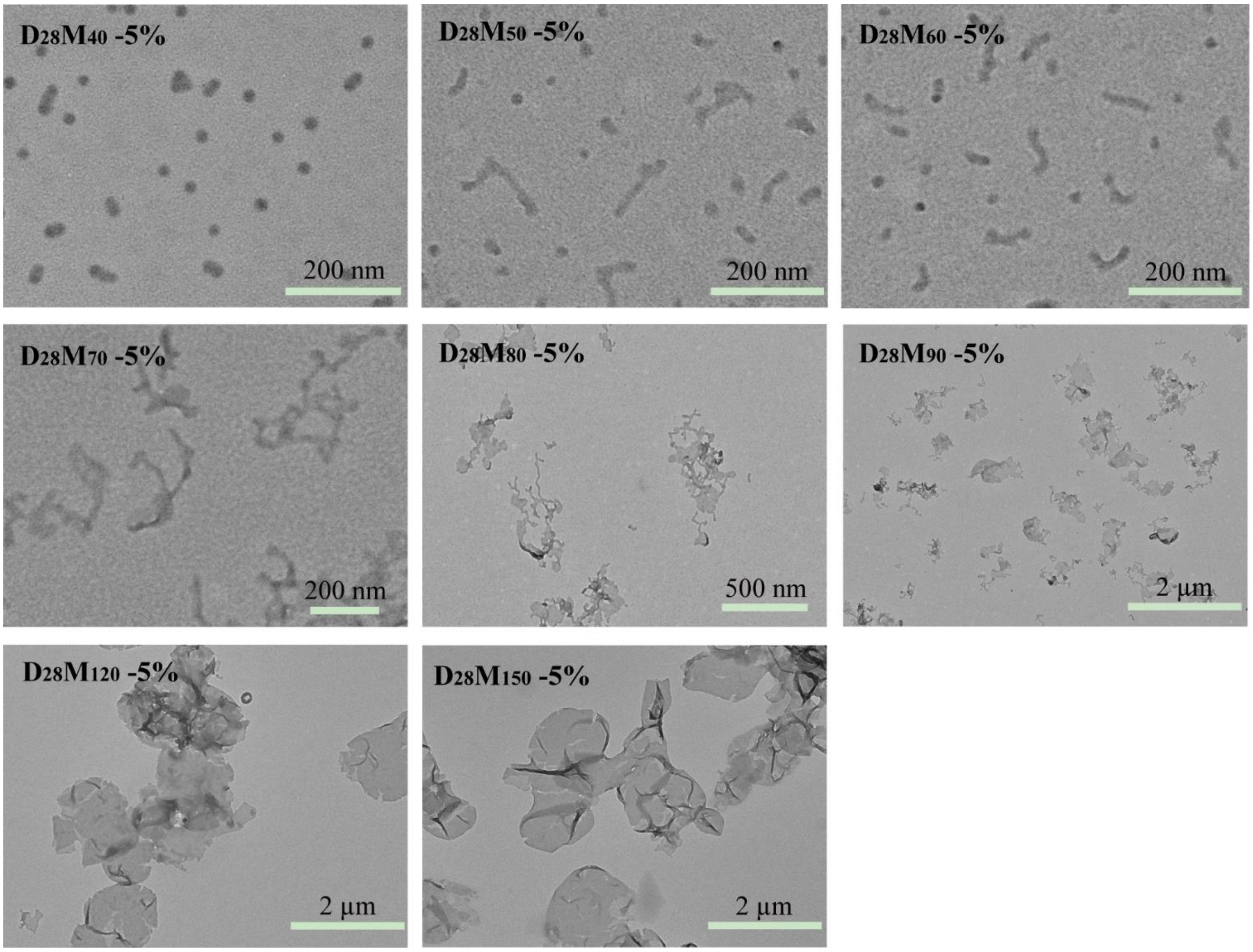

Figure S16. TEM images of $PDMAEMA_{28}$-$PMAIGP_n$ nano-objects with various target degree of polymerization of MAIGP at solids content of 5%, $PDMAEMA_{28}$ as macro-agent at 70 °C, [PDMAEMA]/[AIBN] = 1:0.2.

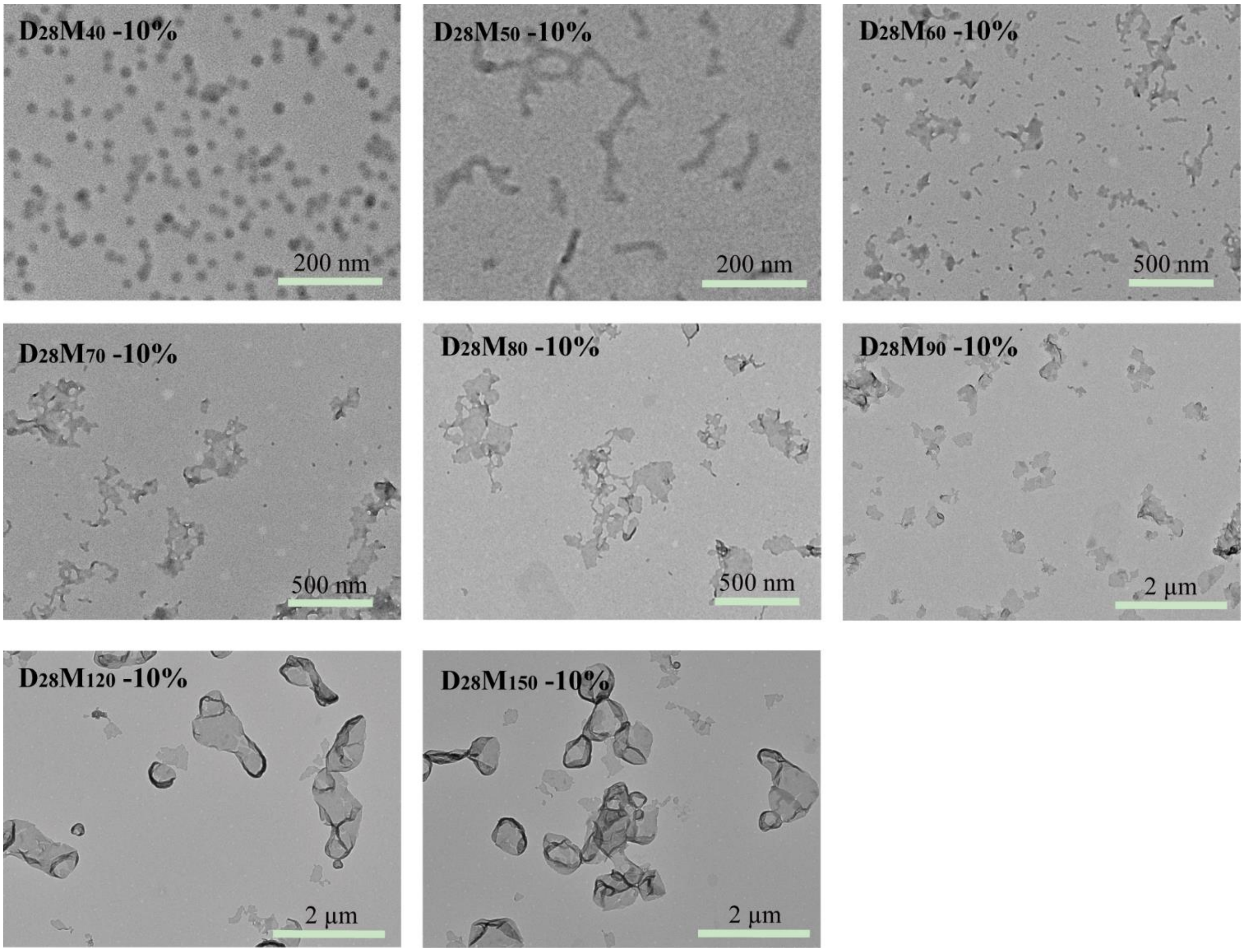

Figure S17. TEM images of $PDMAEMA_{28}$-$PMAIGP_n$ nano-objects with various target degree of polymerization of MAIGP at solids content of 10%, $PDMAEMA_{28}$ as macro-agent at 70 °C, [PDMAEMA]/[AIBN] = 1:0.2.

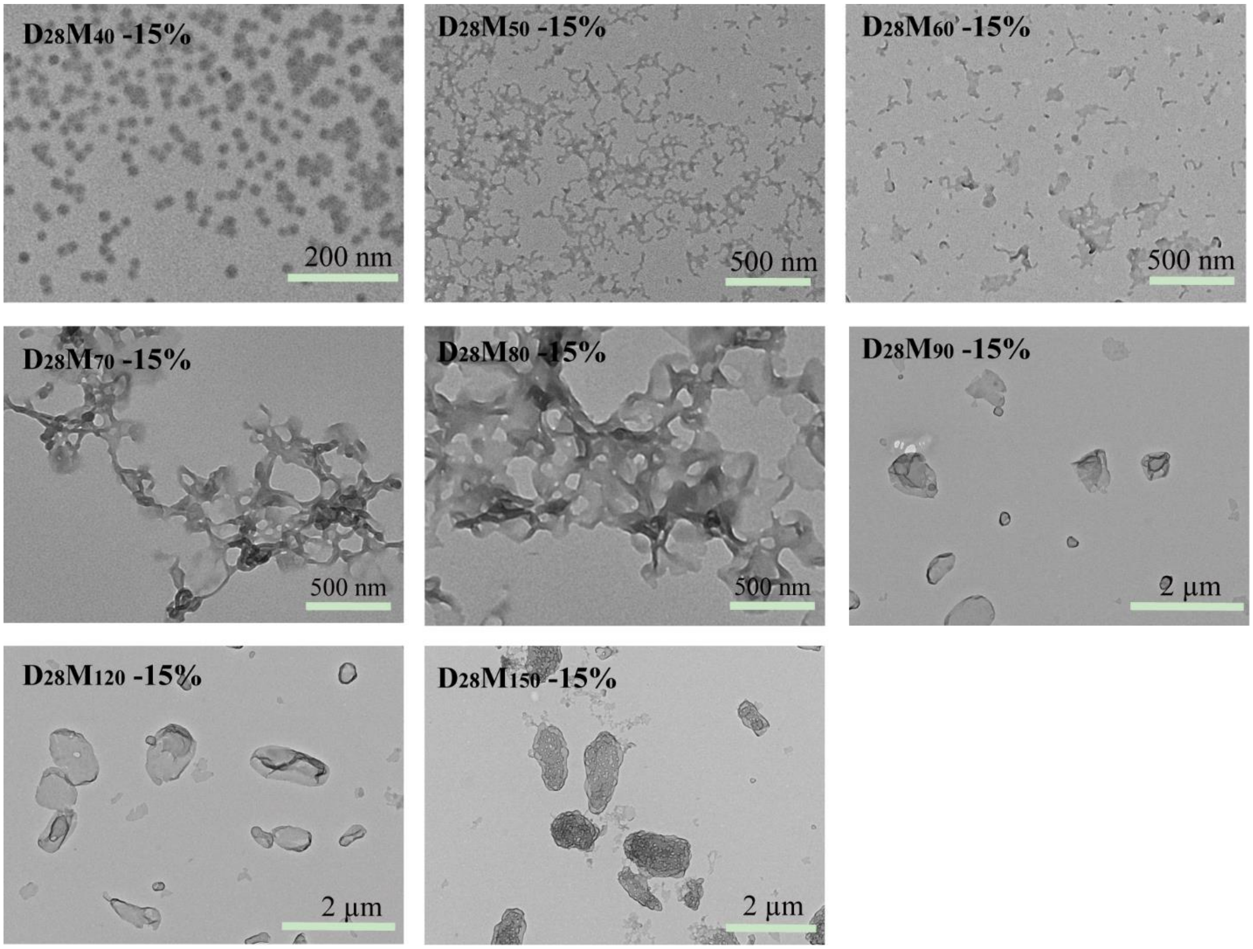

Figure S18. TEM images of PDMAEMA$_{28}$-PMAIGP$_n$ nano-objects with various target degree of polymerization of MAIGP at solids content of 15%, PDMAEMA$_{28}$ as macro-agent at 70 °C, [PDMAEMA]/[AIBN] = 1:0.2.

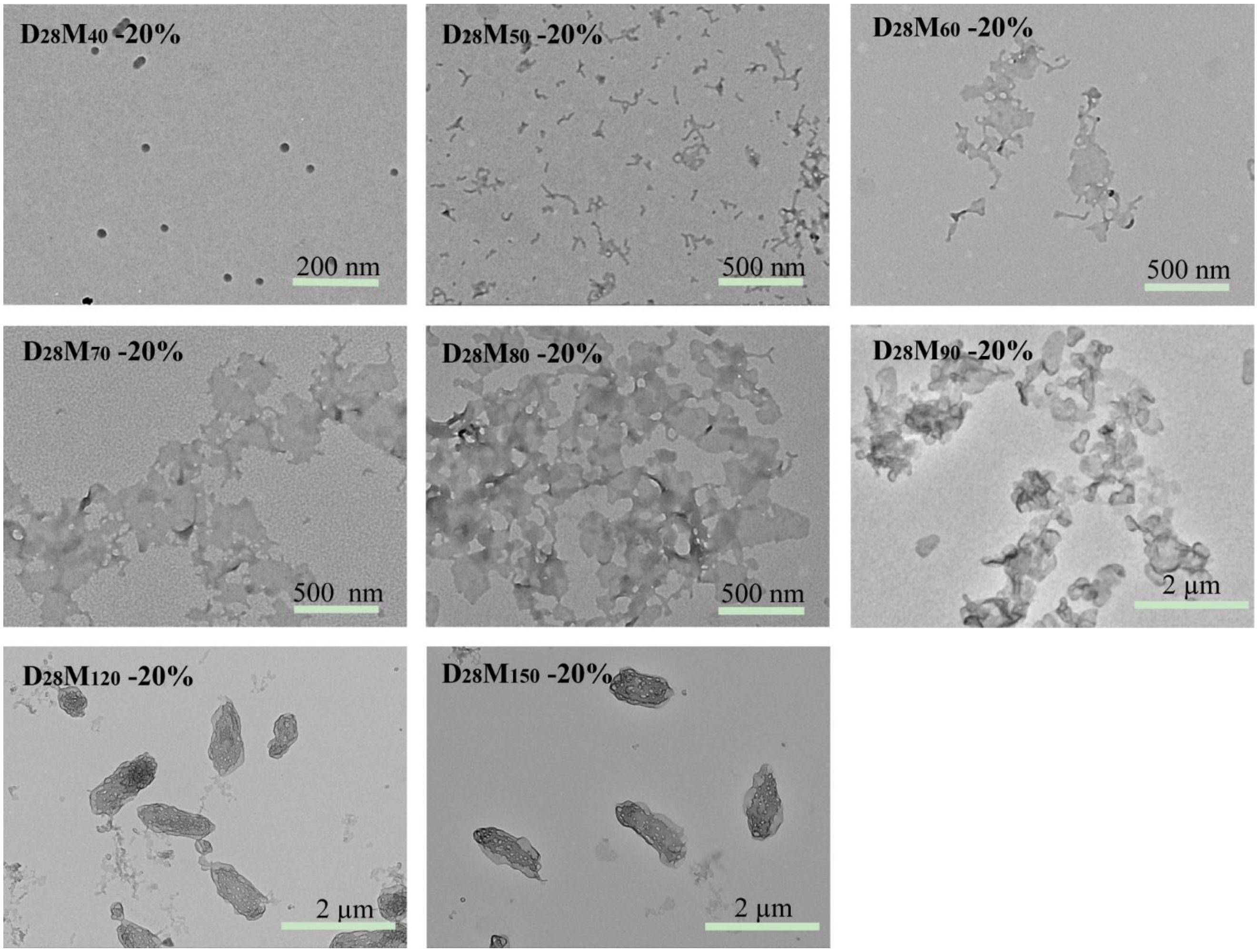

Figure S19. TEM images of $PDMAEMA_{28}$-$PMAIGP_n$ nano-objects with various target degree of polymerization of MAIGP at solids content of 20%, $PDMAEMA_{28}$ as macro-agent at 70 °C, [PDMAEMA]/[AIBN] = 1:0.2.

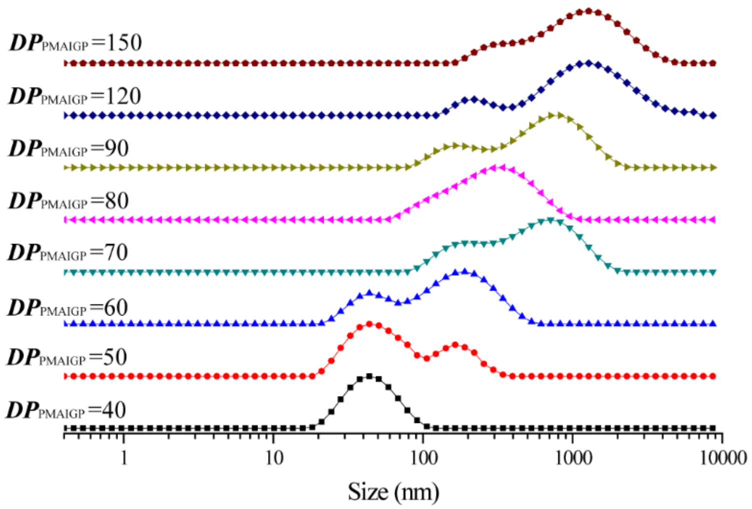

Figure S20. Normalized DLS results of $PDMAEMA_{28}$-$PMAIGP_n$ nano-objects with various target degree of polymerization of MAIGP at solids content of 5%, $PDMAEMA_{28}$ as macro-agent at 70 °C, [PDMAEMA]/[AIBN] = 1:0.2.

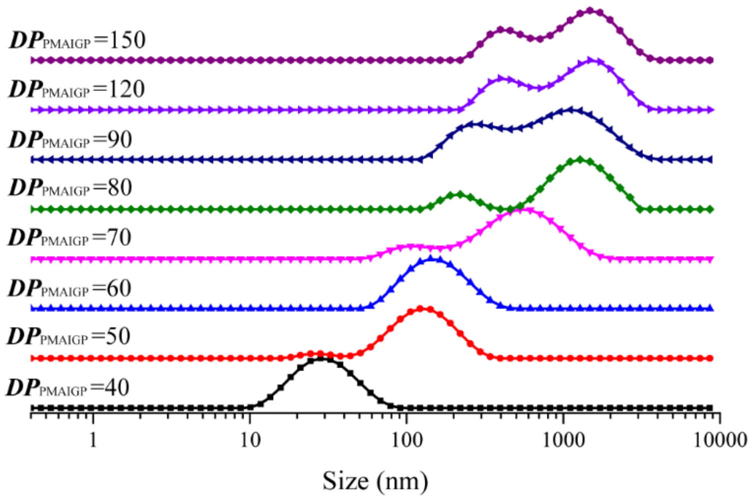

Figure S21. Normalized DLS results of $PDMAEMA_{28}$-$PMAIGP_n$ nano-objects with various target degree of polymerization of MAIGP at solids content of 10%, $PDMAEMA_{28}$ as macro-agent at 70 °C, [PDMAEMA]/[AIBN] = 1:0.2.

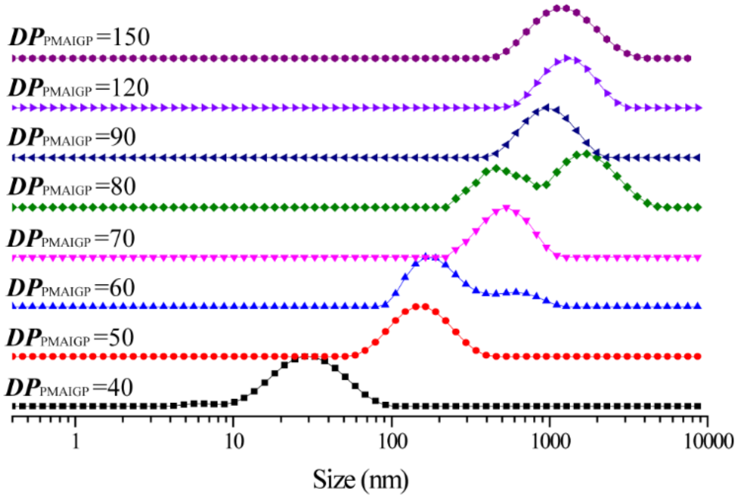

Figure S22. Normalized DLS results of $PDMAEMA_{28}$-$PMAIGP_n$ nano-objects with various target degree of polymerization of MAIGP at solids content of 15%, $PDMAEMA_{28}$ as macro-agent at 70 °C, [PDMAEMA]/[AIBN] = 1:0.2.

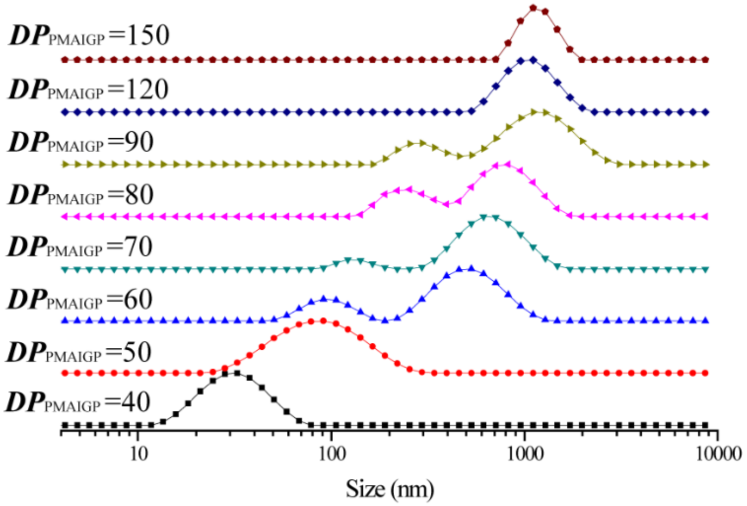

Figure S23. Normalized DLS results of $PDMAEMA_{28}$-$PMAIGP_n$ nano-objects with various target degree of polymerization of MAIGP at solids content of 20%, $PDMAEMA_{28}$ as macro-agent at 70 °C, [PDMAEMA]/[AIBN] = 1:0.2.

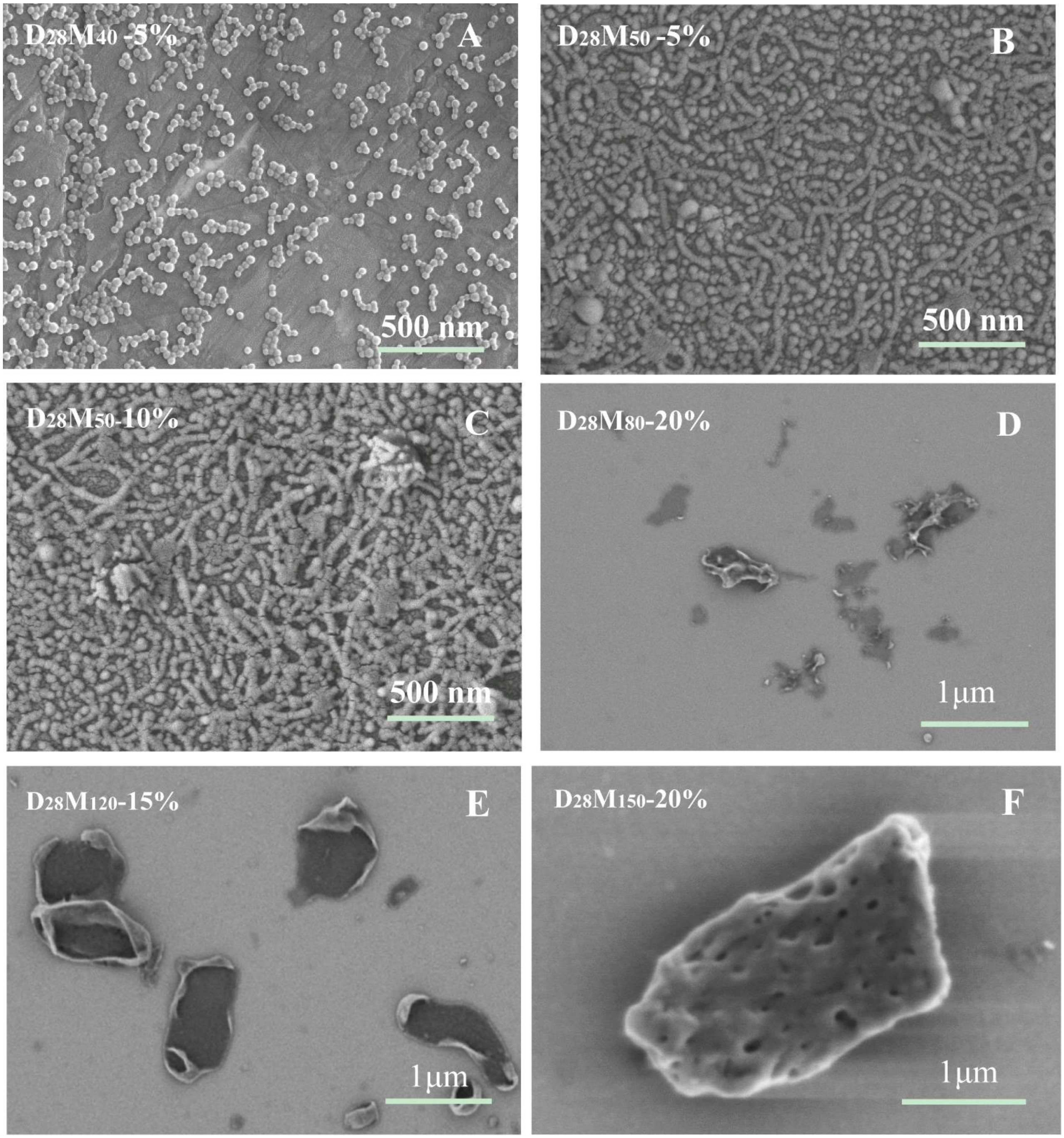

Figure S24. SEM images of representative morphologies of glyco-assemblies. A spheres, B mixture of spheres and linear worms, C branched worms, D lamellae, E multilayer lamellae, F complex vesicles. $PDMAEMA_{28}$ was used as macro-agent at 70 °C, [PDMAEMA]/[AIBN] = 1:0.2.

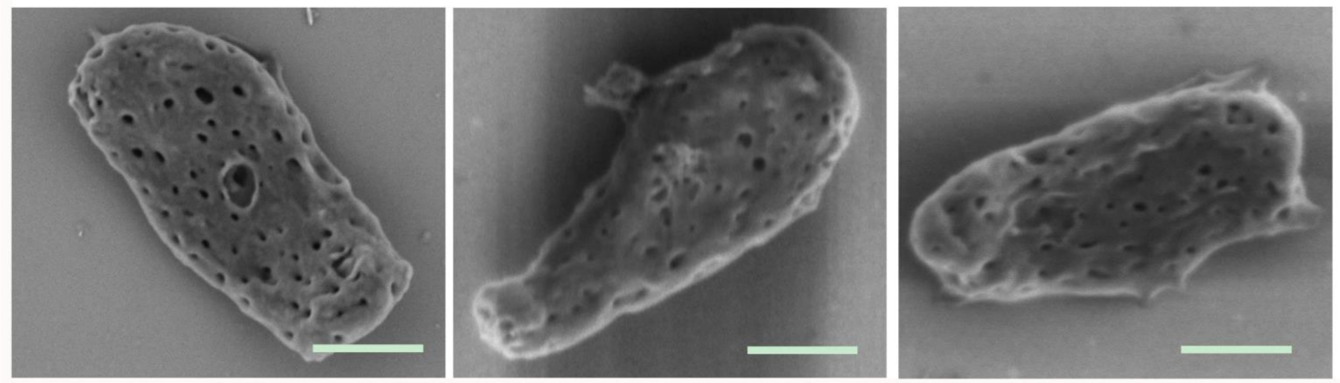

Figure S25. SEM images of different complex vesicles in one sample (scale bar = 500 nm).

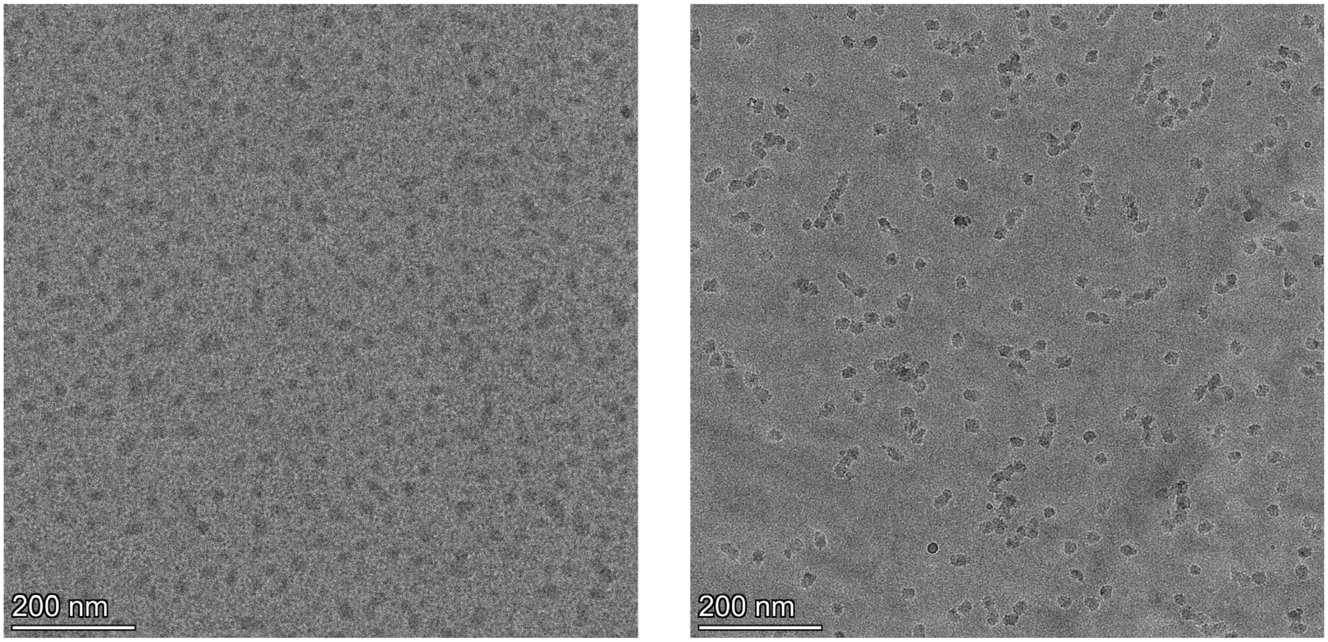

Figure S26. TEM image (left) and cryo-TEM image (right) of micelles ($D_{28}M_{40}$-5%).

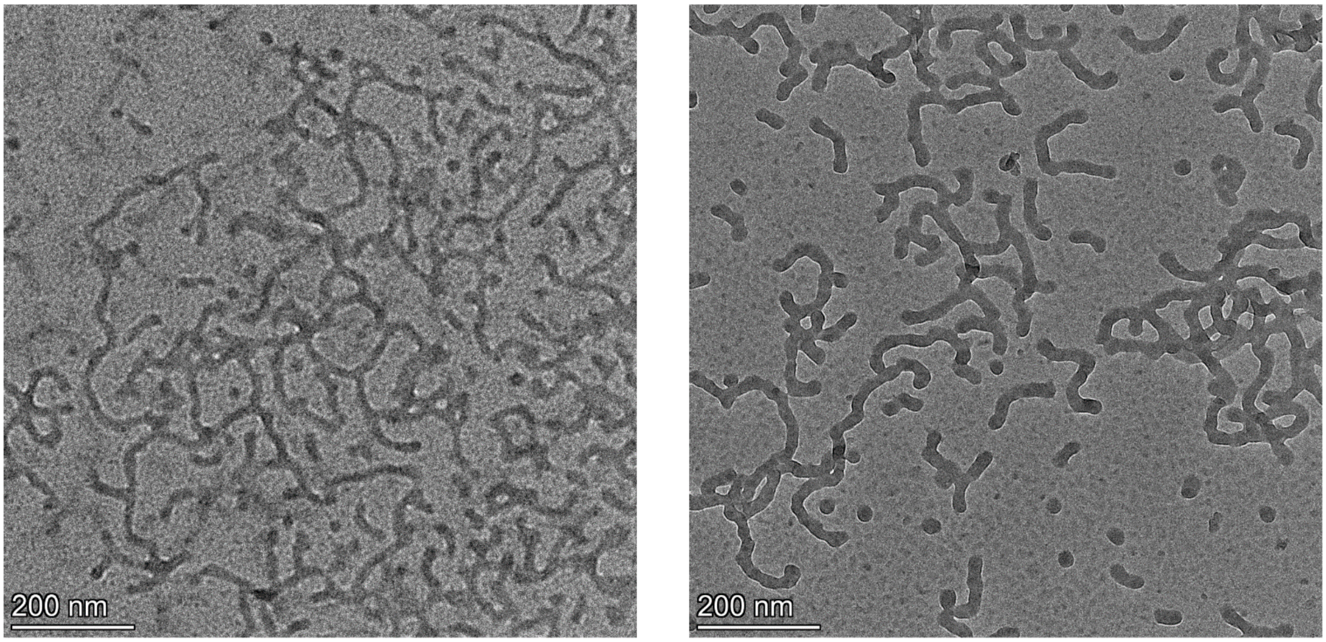

Figure S27. TEM image (left) and cryo-TEM image (right) of branched worms ($D_{28}M_{50}$-10%).

[1] L. Qiu, C. Y. Hong, C. Y. Pan, *Int. J. Nanomed.* **2015**, *10*, 3623-3640.
[2] L. Qiu, J. W. Li, C. Y. Hong, C. Y. Pan, *ACS Appl. Mater. Interfaces* **2017**, *9*, 40887-40897.
[3] D. L. Patton, R. C. Advincula, *Macromolecules* **2006**, *39*, 8674-8683.
